# Characterization of novel *Klebsiella pneumoniae* phages isolated from hospital wastewater in Mumbai, India

**DOI:** 10.3389/fmicb.2026.1953496

**Published:** 2026-10-09

**Authors:** Sofia E. Platova, Roman B. Gorodnichev, Anna V. Shabalina, Veronika A. Osnach, Anastasiia E. Plakhotnik, Maryam O. Sidorskaia, Anastasiia O. Krivulia, Andrey V. Moiseenko, Dmitrii E. Polev, Mariia A. Makarova, Ludmila A. Kraeva, Egor A. Shitikov, Anna S. Dolgova, Vladimir G. Dedkov

**Affiliations:** 1 Saint-Petersburg Pasteur Institute, St. Petersburg, Russia; 2 Lopukhin Federal Research and Clinical Center of Physical-Chemical Medicine of Federal Medical Biological Agency, Moscow, Russia; 3 Faculty of Biology, Lomonosov Moscow State University, Moscow, Russia; 4 North-Western State Medical University named after I.I. Mechnikov, St. Petersburg, Russia; 5 Military Medical Academy named after S.M. Kirov, St. Petersburg, Russia; 6 Martsinovsky Institute of Medical Parasitology, Tropical and Vector Borne Diseases, Sechenov First Moscow State Medical University, Moscow, Russia

**Keywords:** bacteriophage, depolymerase, genome, Klebsiella pneumoniae, novel species, phage therapy, Przondovirus, Teetrevirus

## Abstract

**Introduction:**

The escalating antimicrobial resistance in *Klebsiella pneumoniae* underscores the urgent need for alternative therapies. Phage therapy represents a promising approach owing to its specificity, phage self-replication, and potential synergy with antibiotics. Yet, the narrow host range of phages and rapid emergence of resistance necessitate the discovery of novel phages with diverse specificities, which can be facilitated by screening geographically distant environmental reservoirs. Here, we describe four novel *Klebsiella* bacteriophages belonging to the subfamily *Studiervirinae*, isolated from hospital wastewater in Mumbai, India. We characterized their host range, infection kinetics, physicochemical stability, complete genome sequences, phylogenetic placement, and encoded depolymerases to evaluate their potential as candidates for precision phage therapy against *K. pneumoniae*.

**Methods:**

Phages were isolated via enrichment on clinical *K. pneumoniae* and sequenced using a hybrid approach. Following genomic annotation and *in silico* depolymerase prediction, phages were characterized by transmission electron microscopy, one-step growth curves, and thermal/pH tolerance assays. The capsular specificity of native phages and recombinant depolymerases was evaluated via spot assays against 198 clinical *K. pneumoniae* isolates.

**Results:**

The four phages were classified as novel species within the subfamily *Studiervirinae* (genera *Przondovirus* and *Teetrevirus*), exhibiting strictly lytic lifestyles and favorable safety profiles devoid of antimicrobial resistance genes. They demonstrated strict specificity for clinically prominent capsular types (KL2, KL22, KL25, KL35, and KL47), with phage 1627 uniquely displaying dual specificity for both KL2 and KL22. All phages showed robust stability across broad pH (4–11) and temperature (4–50 °C) ranges. Expression of the recombinant depolymerase yielded preliminary evidence of targeted capsular degradation via spot assays, supporting *in silico* structural predictions of potential *in vitro* lytic activity.

**Discussion:**

These findings suggest that cross-regional environmental screening is a promising strategy for identifying phages effective against local multidrug-resistant clinical pathogens. Moreover, these newly described phage species and their putative depolymerases represent a promising addition to the expanding therapeutic arsenal against *K. pneumoniae* infections.

## Introduction

*Klebsiella pneumoniae* is one of the major causes of healthcare-associated infections, particularly among critically ill and immunocompromised patients (Sati et al., 2025). Currently, *K. pneumoniae* poses a substantial global health burden, having been associated with approximately 800,000 deaths in 2019, of which 80% were attributed to antimicrobial resistance (Song et al., 2025). Notably, over half of these fatal infections were caused by strains resistant to carbapenems and third-generation cephalosporins. In healthcare settings, carbapenemase-producing strains disseminate readily, carrying enzymes such as KPC, NDM, VIM, and OXA-48-type variants, which render these infections challenging to treat (Kumaran et al., 2022). The continued spread of these pathogens, coupled with the diminishing efficacy of conventional antibiotics, underscores the pressing need for novel antibacterial strategies to combat multidrug-resistant *K. pneumoniae*.

As the efficacy of conventional antibiotics wanes, bacteriophages have re-emerged as a promising therapeutic alternative. Bacteriophages offer distinct advantages over conventional antibiotics, including *in situ* self-replication at the site of infection and narrow host specificity, which minimizes disruption to the commensal microbiota (Gordillo Altamirano and Barr, 2019). This specificity renders phages particularly suitable for targeted therapy, but simultaneously constrains the clinical utility of any individual phage, whose activity cannot be reliably predicted against genetically and phenotypically diverse clinical isolates (Łusiak-Szelachowska et al., 2022). Moreover, the narrow host range of bacteriophages and the rapid emergence of bacterial resistance necessitate the continuous discovery of novel phages with diverse host specificities. Clinical implementation of phage therapy therefore relies considerably on curated phage banks comprising large, phenotypically and genomically characterized collections that enable the rapid screening and selection of active phages against individual clinical isolates (Topa-Pila et al., 2026).

Phage-host coevolution can drive local adaptation, generating uncertainty as to whether phages isolated in one region will retain activity against bacterial populations circulating elsewhere (Bleriot et al., 2023). Conversely, phages isolated from distant regions have been successfully employed to treat local strains resistant to both multiple drugs and phages (Yerushalmy et al., 2019). These observations support the cross-regional exchange of well-characterized phages as a means of expanding the functional diversity of therapeutic phage banks; however, they do not imply that phage activity is universally transferable between regions. The genetic and phenotypic composition of *K. pneumoniae* populations varies geographically, encompassing differences in clonal lineage prevalence, surface receptor expression, and intracellular anti-phage defense systems (Liao et al., 2022; Hu et al., 2024; Shamanna et al., 2024; Li et al., 2026; Wacnik et al., 2026). Geographic distance should therefore be regarded not as a direct biological barrier to infection, but rather as a proxy for underlying disparities in relevant host determinants. Ultimately, the practical utility of cross-regional phage sourcing hinges on whether the host-recognition and replication requirements of imported phages align with the surface structures and intracellular environment of the target bacterial population.

In *K. pneumoniae*, one of the principal surface determinants of phage susceptibility is the capsular polysaccharide, which acts both as a physical barrier and as a primary receptor for many lytic phages (Haudiquet et al., 2024). The structural diversity of the *K. pneumoniae* capsule underpins its classification into over 130 capsular (K) types (Wu et al., 2023), also referred to as KL types, when inferred from sequence analysis rather than serologically (Wyres et al., 2016). Given that capsular type distribution varies across populations, differences in this distribution may constrain the transferability of phages between geographical regions. To initiate infection, *K. pneumoniae* phages commonly employ tail fiber- or tailspike-associated depolymerases that recognize and degrade particular capsule structures (Dunstan et al., 2021). While many bacteriophages encode a single depolymerase specific to one capsular type, significant functional diversity exists (Pan et al., 2017; Latka et al., 2019; Liu et al., 2020; Blundell-Hunter et al., 2021). Thus, correspondence between depolymerase specificity and the KL-type composition of a target population may provide a biologically grounded first approximation of cross-regional phage applicability, although it cannot by itself guarantee productive infection at the strain level.

However, despite the potential value of international phage exchange, it remains unclear to what extent geographically distant environmental reservoirs can reliably yield phages active against local clinical isolates, and which molecular determinants underlie such cross-regional compatibility (Martins et al., 2022; Haudiquet et al., 2024). Hospital wastewater has been established as a reservoir for novel therapeutic phages, as it is enriched with diverse bacteria—including antibiotic-resistant clinical strains—that harbor substantial phage diversity (Alharbi and Ziadi, 2021; Abusalah et al., 2025). Leveraging this resource, we screened hospital wastewater from the Effluent Treatment Plant at K.J. Somaiya Hospital & Research Center (Mumbai, India) for phages active against *K. pneumoniae* clinical strains isolated in Moscow and Saint Petersburg, Russia, resulting in the isolation of four novel *Klebsiella* bacteriophages: vB_KpnP-SPI_1047, vB_KpnP-SPI_1627, vB_KpnP-SPI_KBKp12, and vB_KpnP-SPI_G66 (hereinafter referred to as phages 1047, 1627, KBKp12, and G66). We characterized their host range, infection kinetics, physicochemical stability, complete genome sequences, phylogenetic placement, and encoded depolymerases. Specifically, we examined whether a geographically remote wastewater source could yield phages active against Russian clinical isolates, whether their infectivity patterns were determined by bacterial KL type, and whether experimentally tested depolymerases could explain both KL-restricted and cross-KL lytic activity.

## Materials and methods

### Bacterial strains and cultivation

A total of 198 *K. pneumoniae* strains representing 52 capsular types from two collections were used in the study: 67 isolates from the Lopukhin Federal Research and Clinical Center of Physical-Chemical Medicine of Federal Medical Biological Agency (hereinafter referred to as FRCC), 96 isolates from the Saint-Petersburg Pasteur Institute (hereinafter SPI), and 35 strains represented in both collections (Supplementary Table 1). The strains were cultured in lysogeny broth (HiMedia, Mumbai, India) at 37 °C. The capsular type was determined by sequencing the *wzi* gene (Brisse et al., 2013).

### Phage isolation, purification, and storage

A panel of 48 *K. pneumoniae* strains from the FRCC collection was used for bacteriophage isolation (Supplementary Table 1). Wastewater samples collected from K.J. Somaiya Hospital & Research Center [Mumbai, India, (19.04735, 72.87471)] served as the source of phages. Phage isolation was performed using the enrichment culture method as modified by Van Twest and Kropinski (2009). Water samples were centrifuged at 3,000 × *g* for 15 min to remove large suspended particles. The resulting supernatant was collected and filtered through 0.45-μm pore-size syringe filters (Millipore, Burlington, MA, USA) to eliminate bacterial cells. To a 10-mL aliquot of the filtrate, 10 mL of double-strength lysogeny broth (LB) broth and 20 μL of a logarithmic-phase host culture (OD_600_ = 0.3) were added. The mixture was incubated for 18 h at 37 °C with agitation. Following incubation, the lysate was centrifuged at 3,000 × *g* for 15 min, and the supernatant was filtered through a 0.22-μm pore-size filter (Millipore, Burlington, MA, USA) to remove residual bacterial cells and cellular debris. The resulting phage lysate was serially diluted in LB medium and spotted onto Petri dishes containing double-layer agar seeded with the host strain to detect and isolate phages. Three rounds of plaque purification, each followed by reinfection of an exponentially growing host culture, yielded pure bacteriophage suspensions. Phage titers were determined using the double-agar overlay plaque assay (Mazzocco et al., 2009a,b). The obtained phage lysates were stored at + 4 °C.

### Host range determination

The host range of lytic bacteriophages was determined using a spot test assay (Mazzocco et al., 2009a,b). Briefly, 5 mL of molten 0.7% LB agar containing 100 μL of each test bacterial culture was poured onto plates containing 1.5% LB agar as a base layer. Subsequently, a 5-μL drop of the tested phage suspension was spotted onto the solidified agar surface. Phage susceptibility and resistance were assessed based on the formation of clear plaques after overnight incubation at 37 °C. Additionally, efficiency of plating (EOP) assays were performed for all phage-susceptible strains from the FRCC collection (Supplementary Table 1), as described previously (Kutter, 2009). EOP values were calculated as the mean number of plaque-forming units (PFU) obtained on the target bacterial strain divided by the mean PFU count obtained on the reference host strain. When a phage failed to produce discrete plaques but instead formed a turbid clearing zone (halo) on the bacterial lawn that disappeared upon serial dilution, this phenomenon was classified as lysis from without.

### Transmission electron microscopy for morphological characterization

Before transmission electron microscopy (TEM) analysis all phages were concentrated using 50 kDa centrifugal filters (JetBiofil, China). The concentrated phage lysate was centrifuged using a TLA-110 fixed-angle rotor (Optima MAX-XP ultracentrifuge, Beckman Coulter, USA) at 33,900 × *g* for 1 h at 4 °C. The pellet was washed with 0.1 M ammonium acetate (pH = 6.9) and centrifuged at 33,900 × *g* for 1 h at 4 °C. The wash step was repeated 2 more times. After the wash step, the pellet was incubated with 0.1 M ammonium acetate for 3 h at 4 °C prior to resuspension.

Sample suspensions were applied to the glow-discharged carbon support film (EMCN, China) and stained with 1% uranyl acetate solution. Images were acquired with JEM-2100 (JEOL, Japan) transmission electron microscope.

### Adsorption assay and one-step growth curve

An adsorption assay was performed as follows. *K. pneumoniae* host strains were grown to exponential phase (10^8^ CFU/mL) and mixed with bacteriophage at a multiplicity of infection (MOI) of 0.01 and incubated at 30 °C. Aliquots were taken at certain intervals: every 2 min from 0 to 14 min and every 4 min from 14 to 30 min and centrifuged at 14,100 × *g* for 2 min to remove adsorbed phages. The titers of free phages were quantified by plaque assay and the percentage of free phages was determined. The experiments were performed in triplicate.

One-step growth curve analyses were performed as previously described (Kropinski, 2009). Briefly, 9.9 mL of exponential-phase culture (10^8^ CFU/mL) was mixed with 100 μL of phage suspension (10^6^ PFU/mL). The phages were allowed to adsorb for 5 min at 30 °C, after which the mixture was diluted to 10^−2^, 10^−3^ and 10^−4^. The diluted mixtures were incubated at 30 °C with agitation (180 rpm). Aliquots were taken at different time points, and phage titers were determined by spot testing. Independent experiments were repeated 3 times.

Burst size was calculated as the ratio of the final number of released phage particles to the initial number of infected bacterial cells at the end of the latent period.

### Thermal stability and pH tolerance

The phage stability was assessed under various conditions of temperature and pH. Phage lysates (10^7^ PFU/mL) were incubated at 4, 22, 37, 50, 60, and 70 °C for 1 h. The bacteriophage titers were determined by spot test.

The phage stability was monitored over a broad pH spectrum ranging from 2 to 13. The pH of the sterile LB liquid medium was adjusted using HCl and NaOH to pH values of 2, 3, 4, 5, 6, 7, 8, 9, 10, 11, and 12, respectively. 100 μL of phage lysates (10^8^ PFU/mL) was added to 900 μL of liquid media with different pH values and incubated for 1 h at 37 °C. All phage titers were determined by spot test. The temperature and pH stability experiments were conducted in triplicates.

### Phage genomic DNA isolation and whole-genome sequencing

Genomic DNA from pure phage lysates was extracted using Norgen Phage DNA Isolation Kit (Norgen Biotek, Canada) according to the manufacturer's protocol.

The obtained DNA preparations were used for shotgun and long-read sequencing libraries construction. Shotgun libraries were prepared using the NadPrep EZ DNA Library Preparation Kit v2 (Nanodigmbio, China). Sequencing was performed on the DNBSEQ-G50 platform (MGI, China) in paired-end mode with read length of 150 nucleotides. Native Barcoding Kit 96 V14 SQK-NBD114.96 (ONT, Oxford, UK) was used for long reads library construction. Sequencing was performed using the Oxford Nanopore Technologies (ONT) MinION device (Oxford, UK) according to the manufacturer's instructions using a high-accuracy basecalling model.

### Whole-genome assembly, functional annotation, and phylogenetic Analysis

Adapter trimming and removal of low-quality sequences were performed using TrimGalore v0.6.7 (Krueger et al., 2021) for short reads and Porechop v0.2.4 (Bonenfant et al., 2023) for long reads. Long reads were further filtered with Filtlong v0.3.1 (Wick, 2026). Genome assembly was carried out using Flye v2.9.6-b1802 (Kolmogorov et al., 2019). To identify direct terminal repeats (DTRs) at the ends of the genomes, filtered long reads were aligned to the assembled genome using Minimap2 v2.24-r1122 (Li, 2018), the alignments were visualized in Integrative Genomics Viewer (IGV v2.16) (Robinson et al., 2011), and the DTRs were manually curated. Finally, short reads were aligned to the manually curated assemblies using BWA MEM v0.7.17 (Li, 2013), and long reads were aligned using Minimap2. All alignments were visualized in the IGV to manually assess assembly continuity and coverage uniformity, facilitating the identification of potential misassemblies and coverage inconsistencies. Assembly completeness and quality were evaluated using PhageScope (Wang et al., 2023).

CDS prediction and functional annotation were performed using Pharokka v1.7.3 (Bouras et al., 2023), supplemented by Empathi (Boulay et al., 2025), eggNOG-mapper v5.0.2 (Cantalapiedra et al., 2021), and DIAMOND blastp v2.0.14 (Buchfink et al., 2015) against NCBI nr and UniRef100. Functional domain and motif searches in amino acid sequences were performed using InterProScan v5.72-103.0 (Jones et al., 2014). Remote protein homology detection was carried out using HHsearch v3.3.0 (Steinegger et al., 2019). Transmembrane domain prediction was performed using DeepTMHMM (Hallgren et al., 2022). Lifestyle prediction was done with BACPHLIP (Hockenberry and Wilke, 2021). Bacterial virulence and antibiotic resistance genes (AMR) were screened using Pharokka, PhageScope, and PhageLeads (Yukgehnaish et al., 2022). Anti-defense system genes were identified via Defense-Finder v2.0.0 (Tesson et al., 2024). The search for genes with potential depolymerase activity was performed using the DePP (Magill and Skvortsov, 2023) and DepoScope (Concha-Eloko et al., 2024).

Phylogenetic analysis was performed using VipTree, which generates a proteomic tree based on genome-wide tBLASTx similarities (Nishimura et al., 2017). Subsequently, genomic similarity with closely related phages was quantified using the VIRIDIC (Moraru et al., 2020) and taxMyPhage v0.3.4 (Millard et al., 2025) tools.

### Expression, purification, functional and structural characterization of recombinant depolymerases

The coding sequences of the predicted depolymerases were amplified from the DNA of purified phages using primers with endonuclease restriction sites (Supplementary Table 2). The predicted depolymerase candidates are hereafter designated as *dep1047_48, dep1627_49, dep1627_50, depKBKp12_46*, and *depG66_49*, where the numerical suffix indicates the corresponding ORF number within the respective phage genome. PCR products were digested by NdeI and XhoI (*dep1627_49, dep1627_50*, and *depG66_49*) or BamHI and XhoI (*depKBKp12_46* and *dep1047_48*) (New England BioLabs, USA) and cloned into expression vector pGD or pGD SUMO (Dolgova et al., 2026) with an N-terminal histidine tag. Competent *E. coli* Turbo cells (New England BioLabs, USA) were transformed for plasmid propagation and isolation with subsequent Sanger sequencing. Correct constructs were used for *E. coli* BL21(DE3) cells (Evrogen, Russia) transformation, followed by protein expression.

Recombinant *E. coli* BL21 (DE3) cells were grown in LB medium supplemented with kanamycin at 37 °C with agitation (180 rpm) until the optical density at 600 nm reached 0.7. The culture was induced by adding 1 mM IPTG, followed by 18 h induction at 18 °C with agitation (180 rpm). Cultures were pelleted by centrifugation (5000 × *g*, 10 min); resuspended in lysis buffer (50 mM Tris-HCl, 300 mM NaCl, pH 8.0); incubated at 37 °C for 30 min with the addition of 10 mg/mL lysozyme (Sigma-Aldrich, USA), 4 U DNase I (Sigma-Aldrich, USA), and 1 mM MgCl_2_; lysed by 3 cycles of freeze-thawing; and sonicated on ice (10 min with 5 s pulse and 10 s pause). Bacterial lysates were centrifuged (30 min, 10,000 × *g*). Then, the lysates were purified by using His-Spin Protein Miniprep (Zymo Research, USA) according to the manufacturer's protocol. The quality of purified proteins was analyzed by SDS-PAGE and Coomassie blue staining.

The activity of recombinant depolymerases was studied by spot test as was previously described with slight modifications (Verma et al., 2009). Briefly, an LB agar plate supplemented with ampicillin was overlaid with molten soft nutrient agar (0.5%) containing 100 μL of bacterial culture. With the phage (10^8^ PFU/mL) serving as a positive control and elution buffer from the His-Spin Protein Miniprep kit (Zymo Research, USA) serving as a negative control, 10 μL of recombinant depolymerase (300 μg/mL) was spotted onto the plate. After overnight incubation at 37 °C, lytic or semi-clear spots were observed.

Amino acid sequence alignment was performed using SnapGene^®^ v.8.2.2 software (from Dotmatics; available at snapgene.com). Trees were reconstructed using the Maximum Likelihood tree algorithm in MEGA X v10.2.2 software (Stecher et al., 2020). Three-dimensional protein structures were predicted with AlphaFold3.0 (Abramson et al., 2024).

### Statistical analysis and data visualization

Statistical analyses, including calculation of mean values and standard deviations for adsorption assays, one-step growth curve analyses, and thermal and pH stability tests, were performed using Python 3 (package numpy). Graphical illustrations were generated using custom Python 3 scripts (packages seaborn and matplotlib) and R scripts (package circlize). Genome annotation and comparative genomics was visualized using LoVis4u (Egorov and Atkinson, 2025).

## Results

### Morphological characteristics and host range

A panel of 48 *K. pneumoniae* strains representing 28 distinct capsular types (KL) was used for bacteriophage isolation (Supplementary Table 1). Four phages were successfully isolated using the following host strains: vB_KpnP-SPI_1047 on KL1047 (KL25), vB_KpnP-SPI_1627 on KPI 1627 (KL2), vB_KpnP-SPI_KBKp12 on KBKp12 (KL35), and vB_KpnP-SPI_G66 on G66 (KL47).

*K. pneumoniae* phage 1047 produced clear and round plaques with a diameter ≤ 1 mm. The phage 1627 showed plaques with clear centers and turbid edges, about 1–2 mm in diameter, with translucent halos. The phage KBKp12 plaques were clear with a range of size 1-2 mm in diameter with translucent halos. The G66 phage showed clear plaques with a diameter of 1–2 mm (Figure 1).

**Figure 1:**
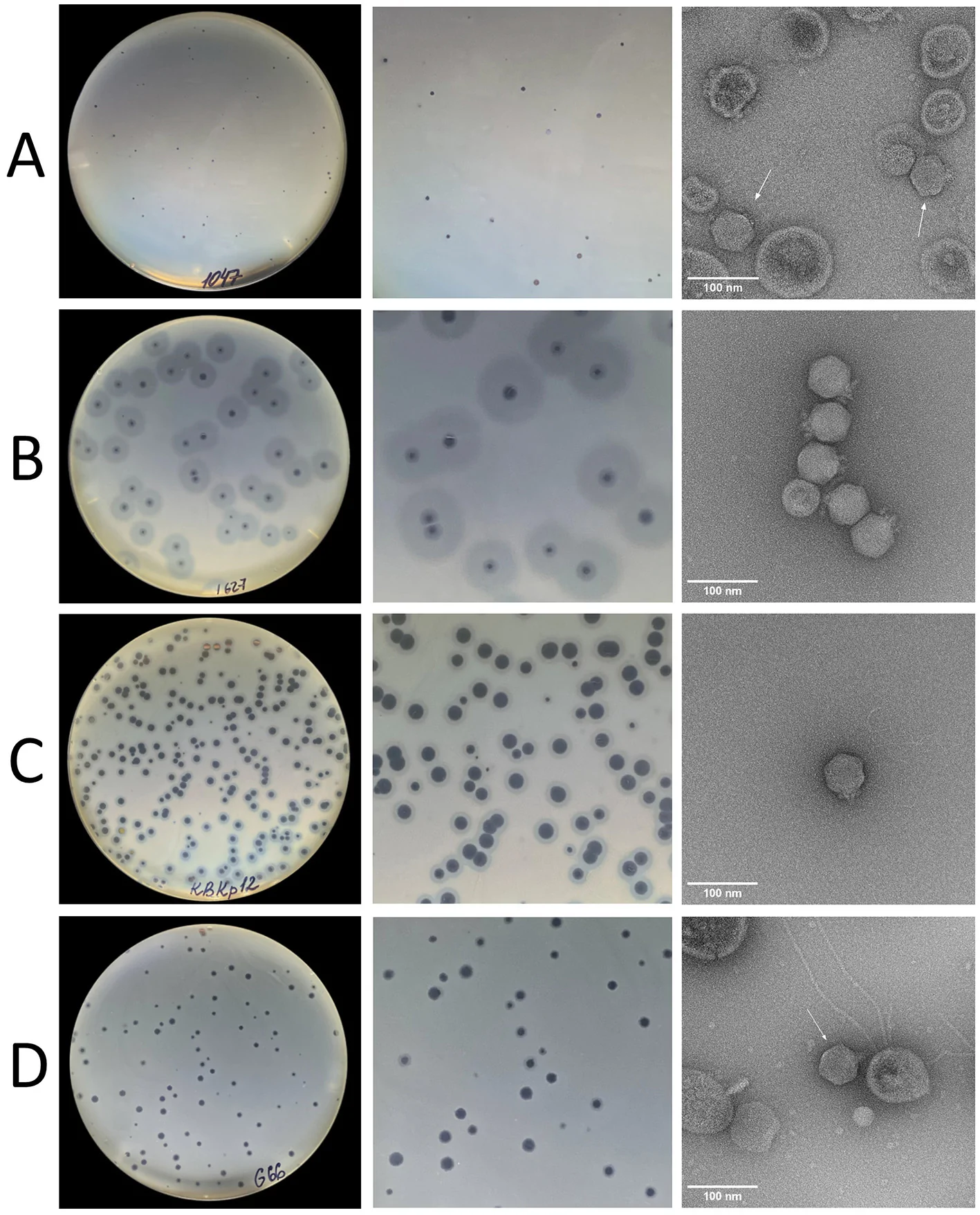
Morphological characterization of four *K. pneumoniae* bacteriophages. Phage plaque morphologies and transmission electron microscopy of **(A)** phage 1047, **(B)** phage 1627, **(C)** phage KBKp12, **(D)** phage G66.

TEM analysis demonstrated that all phages belong to the class *Caudoviricetes*. Phages 1047, 1627, KBKp12, and G66 exhibited morphological features of podoviruses, characterized by an icosahedral head with an approximate diameter of 56 nm, 57 nm, 53 nm, and 55 nm, respectively, and a short tail with an approximate length of 17−8 nm (Figure 1).

Initial screening of all four phages against the primary 102-isolate panel revealed a strong association between phage activity and capsular type. Activity of phages 1047, 1627, KBKp12, and G66 was confined to isolates assigned to KL25, KL2, KL35, and KL47, respectively. Subsequent targeted testing performed in parallel with the recombinant depolymerase assays was consistent with these KL associations and additionally identified phage 1627 activity against KL22 isolates. Of note, a key limitation regarding phage KBKp12 is that variation in susceptibility within the KL35 lineage could not be assessed, as the propagation host was the sole KL35 isolate available in our collection.

Among isolates supporting plaque formation in the primary panel, EOP varied substantially among phage–strain combinations (Figure 2; Supplementary Table 1). Phage 1047 generally retained high plating efficiency on susceptible KL25 isolates, frequently matching or exceeding that observed on its propagation host, whereas phage 1627 displayed heterogeneous plating efficiency among KL2 isolates. In contrast, productive infection by phage G66 was largely restricted to its propagation host and one additional KL47 isolate, with markedly reduced EOP or lysis from without observed on the remaining KL47 strains. Because the only KL35 isolate was also the propagation host of phage KBKp12, its reference EOP value did not permit an independent assessment of plating efficiency across KL35 isolates. Complete strain-level results are provided in Supplementary Table 1.

**Figure 2:**
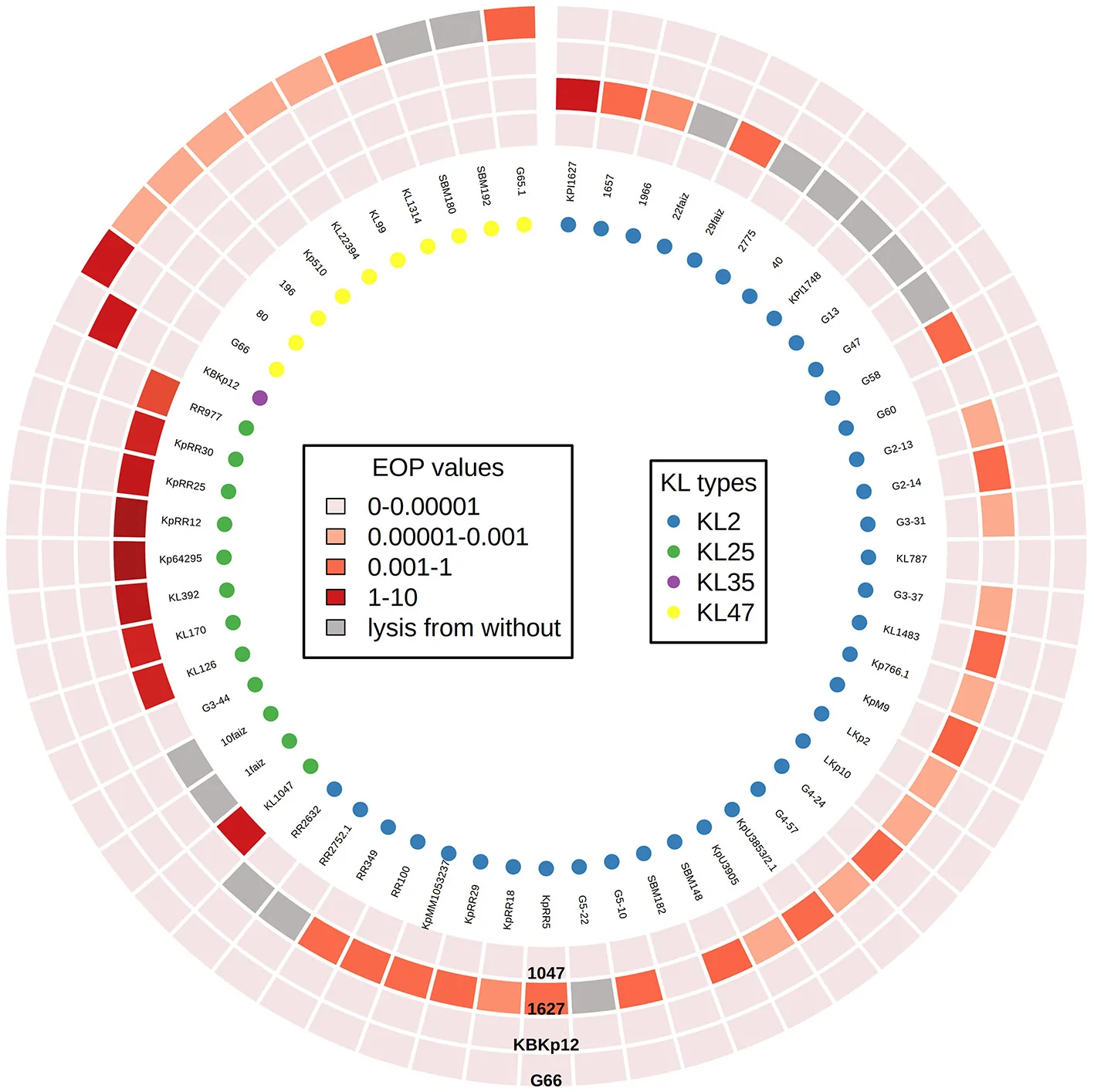
Radial heatmap illustrating the bacteriophage host range and efficiency of plating (EOP). Radial heatmap illustrating the bacteriophage host range and efficiency of plating (EOP). The figure displays *K. pneumoniae* strains representing the four capsular types (KL) targeted by the phages under study, drawn from the FRCC collection. Comprehensive data for all tested strains, including those of other capsular types, are provided in Supplementary Table 1.

### Phage adsorption and growth kinetics, thermal and pH stability

The adsorption curves of the phages 1047 and KBKp12 showed that more than 90% of phages were adsorbed within 4 and 8 min, respectively (Figure 3). The percentage of adsorption of the phage 1627 was around 60% after 12 min of incubation. Studies on the adsorption of the phage G66 have shown that only about 35% of the phage was able to adsorb to the G66 strain by 14 min incubation.

**Figure 3:**
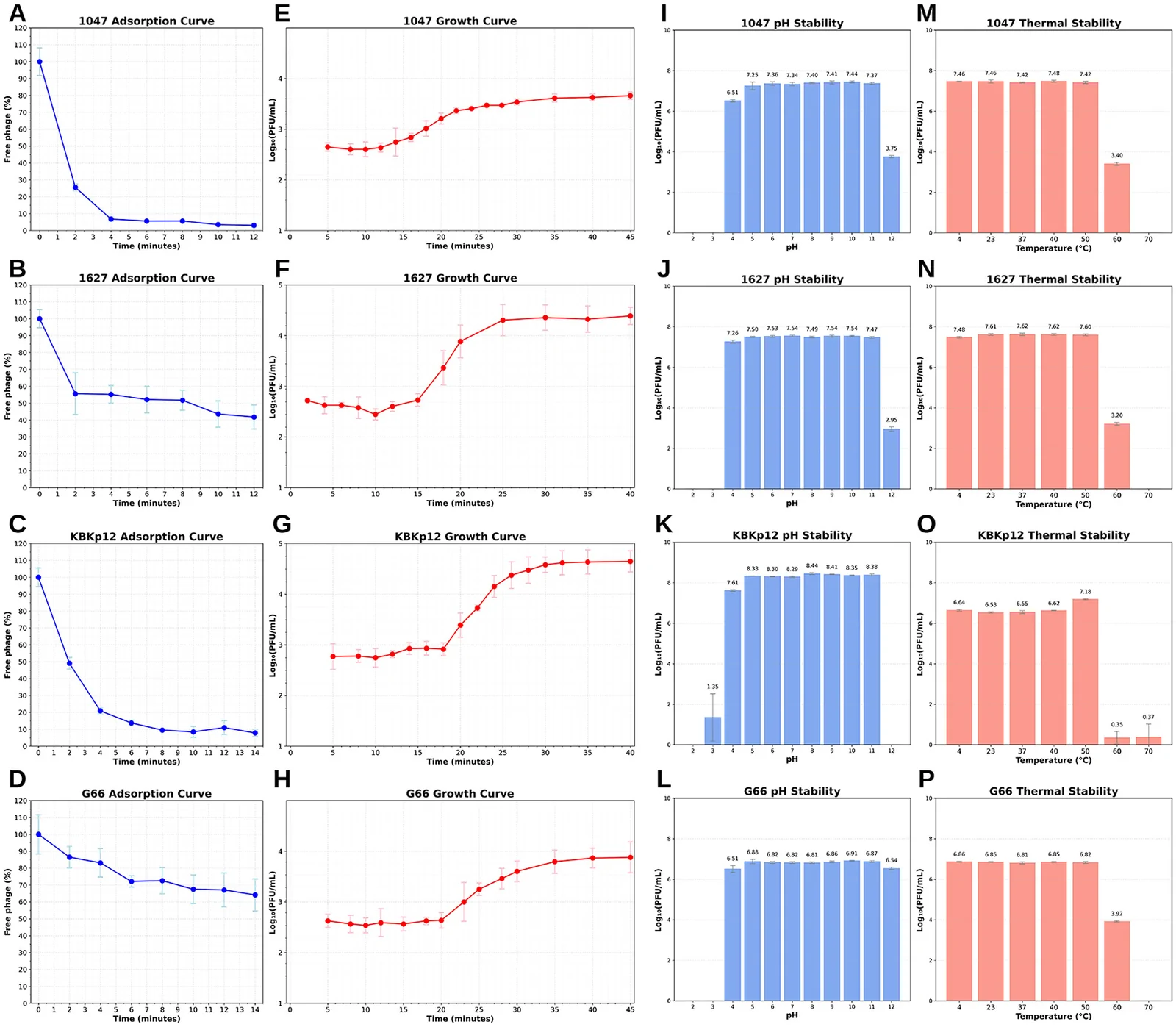
General characterizations of phages 1047, 1627, KBKp12, and G66. **(A, B, C, D)** Adsorption curves of four phages showing the rate of adsorption of each phage. **(E, F, G, H)** One-step growth curves of four phages demonstrating replication dynamics over time. The curves depict the latent, rise, and plateau phases for each phage. **(I, J, K, L)** pH tolerance of the phages. **(M, N, O, P)** Temperature tolerance of the phages. Data represent means from three independent experiments, with error bars indicating standard deviation.

One-step growth assays revealed distinct infection kinetics for each phage (Figure 3). Phage 1047 exhibited the shortest latent period (12 min) but also the smallest burst size (10 PFU/cell). Conversely, phage G66 displayed the longest latent period (20 min) with a moderate burst size of 17 PFU/cell. Phages KBKp12 and 1627 showed intermediate latent periods of 18 and 15 min, respectively; notably, KBKp12 produced the highest burst size (53 PFU/cell), while 1627 yielded 48 PFU/cell.

Thermal stability tests showed that all phages were stable at a wide range of temperatures from 4 °C to 50 °C. However, at a temperature of 60 °C, the titer of the phage KBKp12 drastically decreased; meanwhile, the titers of phages 1047, 1627, and G66 were notably reduced. Complete inactivation of almost all phages occurred at 70 °C after 1 h of incubation. Phage KBKp12 maintained activity after incubation at 70 °C, but its infective capacity was significantly reduced.

Our results demonstrated that all phages had robust tolerance to pH values ranging from 4 to 11, with only slightly changed titers of phages KBKp12 and 1047 at pH 4. However, at pH 12, the phages demonstrated different stability: phage G66 remained stable, the titers of phages 1627 and 1047 were reduced significantly, and phage KBKp12 was completely inactivated. Moreover, at pH 3, the titer of phage KBKp12 drastically decreased, and phages 1047, 1627, and G66 were inactivated. All phages were completely inactivated after 1 h of incubation at pH 2 (Figure 3).

### Genomic characterization and phylogenetic placement

High-quality genomic DNA was extracted from DNase-treated phage lysates and sequenced using a hybrid short- and long-read approach. This strategy yielded four complete, gap-free genomes with high coverage across their entire length. All four phages assembled into single contigs of approximately 40 kbp, with 100% completeness confirmed by quality assessment. Long-read sequencing enabled precise resolution of DTRs at both genome termini. Key genomic features, including DTRs lengths, GC content and the number of predicted open reading frames (ORFs), are summarized in Figure 4.

**Figure 4:**
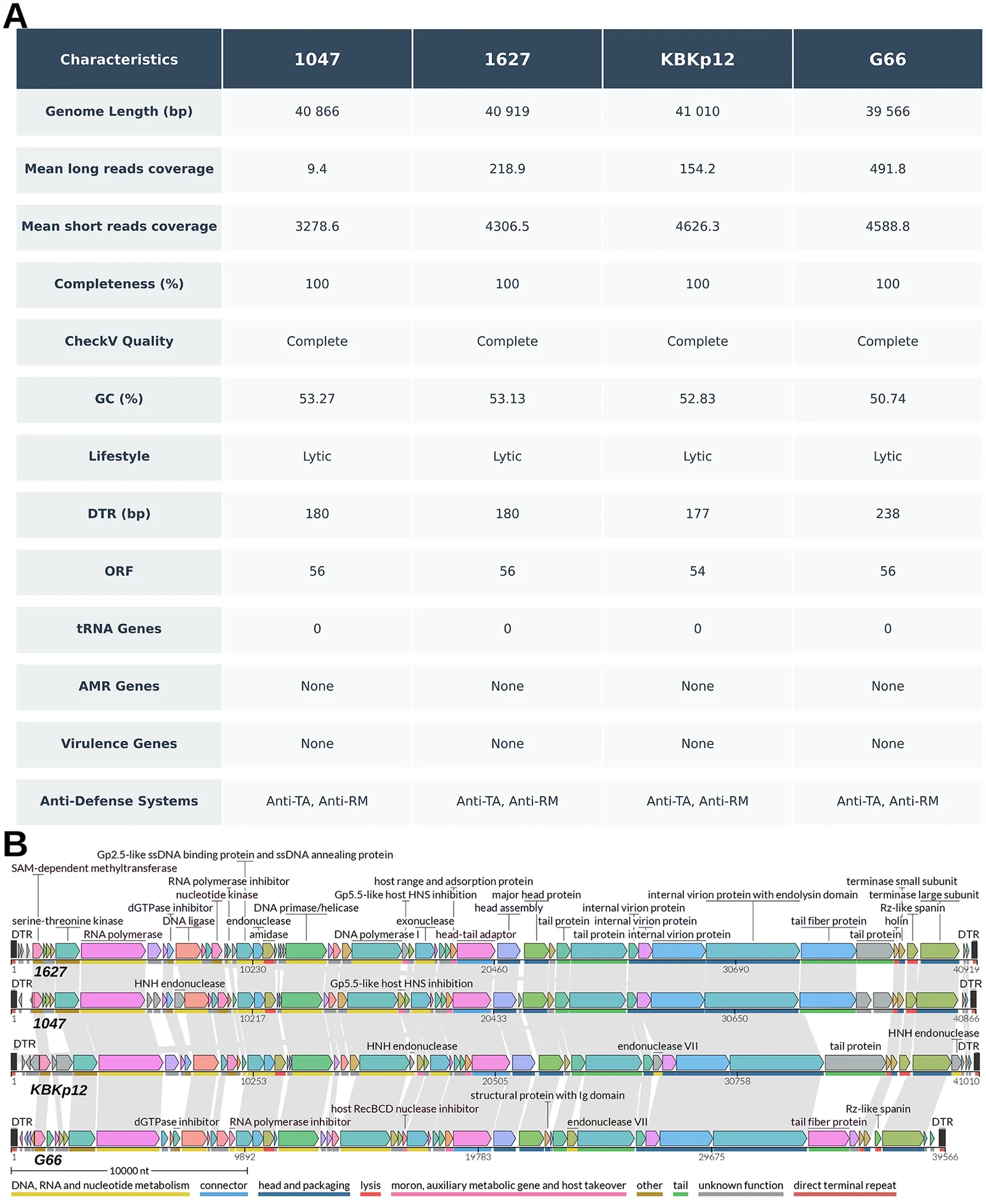
Overview of sequencing, genome assembly, and structural annotation of bacteriophage genomes. **(A)** Summary of genome characteristics and assembly quality metrics. **(B)** Functional annotation and genomic organization. Gray shading indicates sequences sharing ≥ 25% identity over ≥ 80% of their length.

Notably, the vast majority of predicted coding sequences are encoded on the forward DNA strand. Functional annotation enabled putative functional assignment for most ORFs. Comprehensive protein-level annotations for all phage-encoded genes are provided in Supplementary Table 3. The genomes encode a phage-type DNA-directed RNA polymerase and a DNA-directed DNA polymerase I. Genes involved in nucleic acid metabolism and those encoding structural proteins were organized into discrete genomic clusters. Genomic organization and protein arrangement exhibit notable conservation across all four phages, with only minor variations (Figure 4B). A strictly lytic lifestyle was predicted for all phages. Furthermore, no antimicrobial resistance (AMR) or bacterial virulence factors were detected in any of the genomes. All phages were found to encode anti-defense systems, including inhibitors of Toxin-Antitoxin (Anti-TA) and Restriction-Modification (Anti-RM) systems. Transmembrane domain prediction identified a small number of membrane proteins, primarily holins and Rz-like spanins, with the notable exception of the Rz-like spanin in phage G66, which lacked a predicted transmembrane domain. No transmembrane domains were detected in proteins involved in DNA replication, transcription, or metabolism, consistent with their cytoplasmic functions.

Phylogenetic analysis using VipTree assigned phages 1047, 1627, KBKp12, and G66 to the order *Autographivirales* and the family *Autographiviridae* (Figure 5A), which are legacy taxonomic designations corresponding to the family *Autotranscriptaviridae* in the current ICTV taxonomy. Subsequent taxonomic classification using taxMyPhage, which references the ICTV database of dsDNA phages, assigned all four phages to the order *Autographivirales*, family *Autotranscriptaviridae*, subfamily *Studiervirinae* (according to current ICTV taxonomy). Phages 1047, 1627, and KBKp12 were assigned to the genus *Przondovirus*, and phage G66 to the genus *Teetrevirus*.

**Figure 5:**
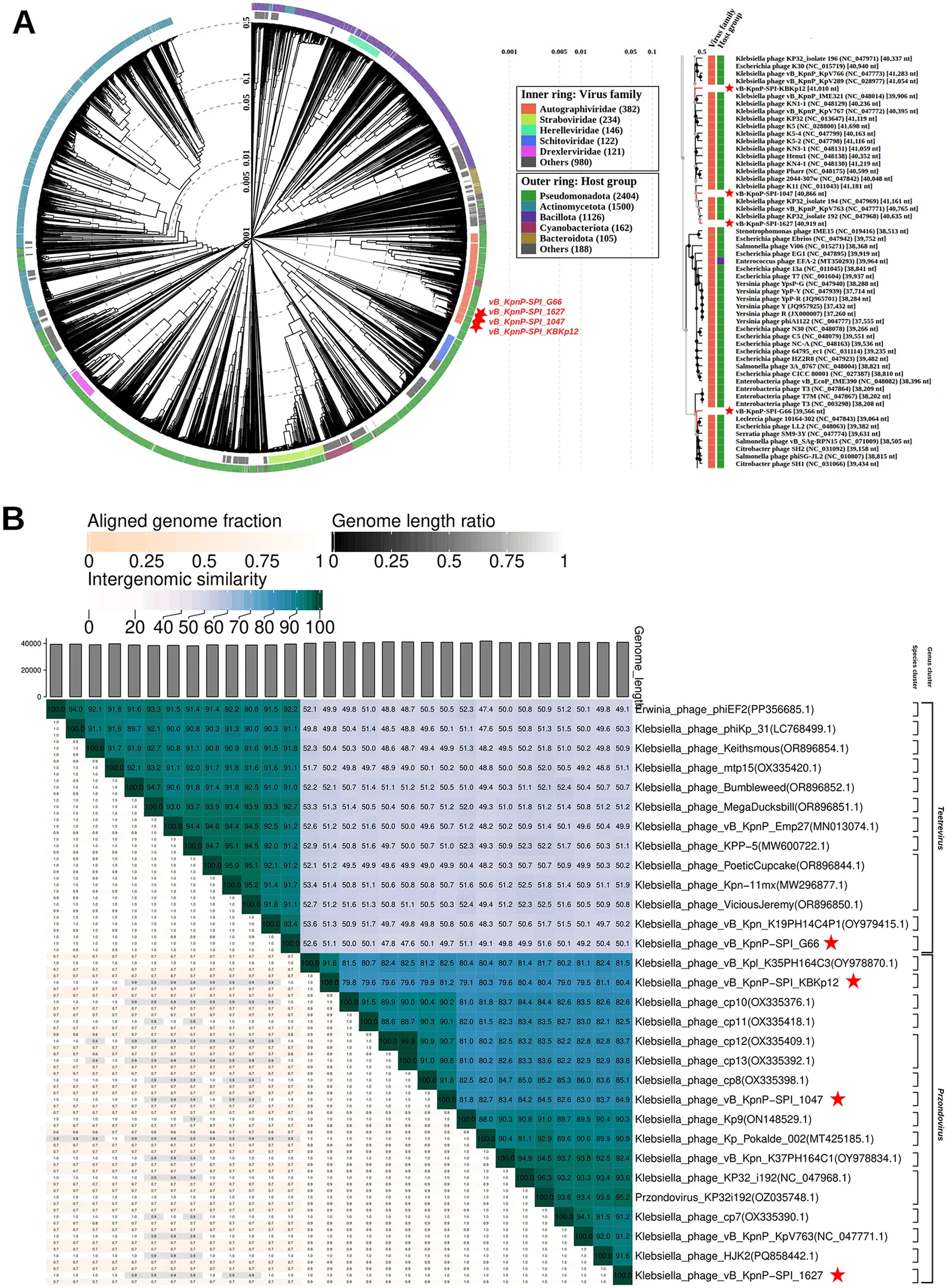
Phylogenetic and genomic relatedness analysis of phages 1047, 1627, KBKp12, and G66. **(A)** Phylogenetic tree of four isolated phages with reference dsDNA bacteriophages constructed using VipTree. The query phages are highlighted in red. **(B)** Pairwise intergenomic similarity analysis calculated using VIRIDIC for phages belonging to the genera *Przondovirus* and *Teetrevirus*. Heatmaps display the closest characterized relatives from GenBank, with only genomes showing ≥ 90% nucleotide identity to the query phages included. Novel phages isolated in this study are indicated by red labels.

To validate species-level demarcation, intergenomic similarity was assessed using VIRIDIC against all publicly available genomes of the respective genera in NCBI GenBank. This tool clusters prokaryotic viruses into species based on a 95% nucleotide identity threshold, requiring > 95% similarity with all representative strains of an existing species; otherwise, the virus is classified as a novel species. None of the isolates shared > 95% nucleotide identity with any previously characterized species, and VIRIDIC-based clustering placed each isolate into a distinct, species-level cluster (Figure 5B). Pairwise intergenomic similarity analysis revealed that phage 1047 shared the highest nucleotide identity (91.8%) with *Klebsiella* phage cp8 (OX335398.1). Analysis revealed that *Przondovirus* KP32i192 (OZ035748.1) is closely related to *Klebsiella* phage KP32_isolate 192 (NC_047968.1; MH172261), the sole exemplar strain of this species in the ICTV database, sharing > 95% mutual nucleotide identity. Phage 1627 exhibits high similarity to this group, with 95.2% nucleotide identity to OZ035748.1 and 93.6% to the exemplar strain MH172261. Although the pairwise identity with the isolate OZ035748.1 exceeds the ICTV species demarcation threshold (> 95%), our assessment based on the genomic clustering indicates that phage 1627 constitutes a distinct species relative to the official exemplar strain. Notably, the close genetic proximity among these isolates suggests the existence of a species complex with blurred taxonomic boundaries within the genus. Phage KBKp12 showed the highest similarity (91.6%) to *Klebsiella* phage vB_Kpl_K35PH164C3 (OY978870.1). Finally, phage G66 is most closely related to *Klebsiella* phage vB_Kpn_K19PH14C4P1 (OY979415.1), with a nucleotide identity of 93.4%.

### Depolymerases

Genome annotation of phages 1047, 1627, KBKp12, and G66 identified putative tail fiber/spike protein-encoding genes: *1047_cds0048* (*vB_KpnP-SPI_1047_CDS_0048*), *1627_cds0049* and *1627_cds0050* (*vB_KpnP-SPI_1627_CDS_0049* and *vB_KpnP-SPI_1627_CDS_0050*, respectively), *KBKp12_cds0046* (*vB_KpnP-SPI_KBKp12_CDS_0046*), and *G66_cds0049* (*vB_KpnP-SPI_G66_CDS_0049*) (Supplementary Table 3). To confirm whether the predicted proteins have polysaccharide-degrading activity, the abovementioned genes were cloned into expression vectors pGD. Recombinant depolymerases Dep1047_48, Dep1627_49, and Dep1627_50 were expressed in *E. coli* BL21 cells and purified (Supplementary Figure 1). The attempts to purify proteins DepKBKp12_46 and DepG66_49 synthesized using expression vectors based on pGD had failed. Therefore, expression plasmids containing a SUMO tag were constructed, with subsequent successful expression and purification of the recombinant proteins DepKBKp12_46 and DepG66_49, which remained in SUMO fusion forms for the activity test (Supplementary Figure 1).

The depolymerase activities of all these recombinant proteins were examined using a spot test on the host strains of the phages and a panel of strains representing other KL-types from the SPI collection to detect translucent spots (Supplementary Table 1). The protein Dep1047_48 only showed activity against the KL25-type strains. The recombinant proteins Dep1627_49 and Dep1627_50 formed semi-clear spots on the KL2-type strains. Moreover, Dep1627_49 also formed translucent spots on the KL22-type strains. DepKBKp12_46 formed semi-clear spots only on the KL35-type host strain (Table 1; Supplementary Figure 2). DepG66_49 didn't demonstrate any activity against tested strains of *K. pneumoniae*, including its host strain.

**Table 1:** Positive results of spot test for phages and their predicted depolymerases on Pasteur Institute's collection of *K. pneumoniae*.

*K. pneumoniae* strain	KL-type	1047	Dep1047_48	1627	Dep1627_49 ^**^	Dep1627–50 ^**^	KBKp12	DepKBKp12_46	G66	DepG66_49
EI1601	KL25	+	+							
KL1047^*^	KL25	+	+							
1faiz	KL25	+	+							
Kp40	KL2			+	+	++				
Kp231	KL2			+	++	+				
KPI 1627^*^	KL2			+	++	+				
1	KL2			+	+	++				
6	KL2			+	+	++				
23	KL2			+	+	++				
67	KL2			+	+	++				
138	KL2			+	++	+				
146	KL2			+	+	++				
186	KL2			+	++	+				
396	KL2			+	++	+				
430	KL2			+	+	++				
476	KL2			+	++	+				
480	KL2			+	++	+				
537	KL2			+	+	++				
766	KL2			+	+	++				
830	KL2			+	+	++				
913	KL2			+	+	++				
925	KL2			+	+	++				
933	KL2			+	++	+				
2399	KL2			+	+	++				
M3	KL2			+	+	++				
M9	KL2			+	+	++				
2104	KL2			+	+	++				
76	KL2			+	+	++				
77	KL2			+	+	++				
1402	KL2			+	++	+				
68	KL2			+	+	++				
81	KL2			+	+	++				
G13	KL2			+	+	++				
EI1939	KL22			+	++					
EI1940	KL22			+	++					
KBKp12^*^	KL35						+	+		
G66^*^	KL47								+	
196	KL47								+	

^*^–*K. pneumoniae* strains used for phage isolation. ^**^–For these depolymerases positive results were assigned based on intensity: the clearly visible halo was marked by “++”, while the weaker one was marked “+”. Comprehensive data for all tested strains, including those with negative results, are provided in Supplementary Table 1.

AlphaFold 3 predicted the structures of all depolymerases, except for the putative protein DepG66_49, with high confidence (Supplementary Figures 3, 4). Additionally, homologous depolymerase sequences were identified and analyzed to assess evolutionary relationships (Supplementary Figure 5).

## Discussion

Aiming to expand the therapeutic arsenal against *K. pneumoniae* strains, we explored hospital wastewater in a geographically distant location. This cross-regional approach is grounded in the persistent coevolutionary arms race between bacteria and bacteriophages. In natural environments, the continuous reciprocal evolution of bacterial defense mechanisms and phage counter-defenses drives local adaptation, which can ultimately diminish the clinical efficacy of locally isolated phages against regional pathogens. By sourcing phages from an ecologically and geographically distinct reservoir, we sought to bypass the specific resistance profiles of local bacterial populations. Consequently, we successfully isolated and characterized four novel bacteriophage species that demonstrated robust lytic activity against Russian clinical isolates. These findings support the concept that cross-regional sampling can be an effective strategy for identifying phages to overcome local bacterial defenses and expand the global phage therapy toolkit.

Morphological, genomic, and phylogenetic analyses confirmed that the four phages are dsDNA tailed phages belonging to the subfamily *Studiervirinae*, classified into the genera *Przondovirus* (1047, 1627, KBKp12) and *Teetrevirus* (G66), representing novel, previously uncharacterized viral species. Functionally, these newly described species exhibited distinct host recognition patterns governed by their capsular specificity. While three phages demonstrated specificity to a single KL type (KL25, KL35, and KL47, respectively), one demonstrated dual specificity and was capable of lysing *Klebsiella* strains of KL2 and KL22. This aligns with the well-established role of the capsular polysaccharide as a primary receptor and physical barrier during phage adsorption. The therapeutic relevance of this specificity is amplified by the clinical prominence of these KL types (Huang et al., 2022; Wang et al., 2025). KL2 is traditionally associated with hypervirulent *K. pneumoniae* lineages, whereas KL47 and KL25 are frequently identified among carbapenem-resistant clinical isolates. According to recent epidemiological surveys (Kang et al., 2023), KL35, KL2, KL47, and KL25 rank among the most prevalent capsular types in public sequence databases, further supporting the broad applicability of these phages. Furthermore, our results demonstrate that the isolated phages are capable of effectively lysing *K. pneumoniae* strains exhibiting multidrug resistance (MDR) to antibiotics from different classes, including third-generation cephalosporins (ceftriaxone, ceftazidime), aminoglycosides (gentamicin), fluoroquinolones (ciprofloxacin), and tetracyclines (Supplementary Table 1), underscoring their significant therapeutic potential.

A primary concern associated with any newly isolated bacteriophage is the uncertainty regarding its safety and the potential risk of horizontal transfer of genes encoding bacterial virulence factors, or antimicrobial resistance determinants to bacterial hosts. Comprehensive genome annotation provides a critical first line of safety evaluation. In this study, we employed an integrative approach for functional annotation of phage proteins, combining predictions from multiple tools and databases. Although a substantial proportion of proteins were annotated as hypothetical, which is a well-known challenge in phage genome annotation (Van Den Bossche et al., 2014), none of the four phage genomes harbored known genes encoding antimicrobial resistance (AMR) determinants, bacterial virulence factors, defense systems, or phage integrases. While the presence of hypothetical proteins precludes definitive safety claims, the absence of these known risk factors suggests a favorable genomic profile and supports the potential suitability of these phages for therapeutic applications (Supplementary Table 3).

Additionally, during the annotation stage, we applied *in silico* methods to predict phage-encoded proteins with potential depolymerase activity. By integrating predictions from two neural network-based tools (Magill and Skvortsov, 2023; Concha-Eloko et al., 2024) with protein domain searches and remote homology detection, we identified putative depolymerases in three of the four phages (Supplementary Table 3). For phage G66, no protein exhibited high-confidence depolymerase predictions and distinctive domain/remote homology signatures. However, *vB_KpnP-SPI_G66_CDS_0049* (tail fiber protein) was selected as the most plausible depolymerase candidate based on its genomic position and sequence homology with tail fiber proteins known to harbor depolymerase domains in related phages (Lukianova et al., 2026). The three-dimensional structures of the putative depolymerases were predicted using AlphaFold 3. With the exception of DepG66_49, all modeled proteins exhibited well-resolved structural domains characteristic of this enzyme family, including the N-terminal domain, C-terminal domain, and catalytic domain (Supplementary Figures 3, 4).

The *in silico* predictions were corroborated by subsequent experimental observations. Specifically, using a panel of *Klebsiella* strains from the SPI collection, we assessed the lytic activity of the isolated phages, while spot assays supported their depolymerase activity (Supplementary Table 1). Of particular interest is the specificity profile of phage 1627. Beyond its primary host, screening revealed additional lytic activity against the KL22 capsular type, while its depolymerases demonstrated activity toward *Klebsiella* strains belonging to both the KL2 and KL22 capsular types. Although both Dep1627_49 and Dep1627_50 exhibited activity against KL2-type strains, only Dep1627_49 was active against KL22. This presents a counterintuitive scenario: the phage's ability to degrade two distinct capsule types is not mediated by two depolymerases with complementary specificities; rather, one protein targets both capsule types, while the other targets only one. Extended depolymerase K-type specificities are documented for *Klebsiella* phages (Domingo-Calap et al., 2020); however, this phenomenon typically involves capsules with similar structures (Cheetham et al., 2024). Thus, the dual specificity of Dep1627_49 toward the unrelated KL2 and KL22 types is notable. We speculate that these structurally distinct capsular polysaccharides may share a common three-dimensional or electrostatic motif recognized by the depolymerase, although a detailed investigation of this mechanism is beyond the scope of the present study. Conversely, complementary specificity of two distinct depolymerases toward *Klebsiella* strains belonging to the same capsule type has also been documented (Liu et al., 2020; Cheetham et al., 2024). While Dep1627_49 and Dep1627_50 shared an identical host range within the KL2 serotype (Table 1), quantitative differences in lysis efficiency were observed for each strain tested (Supplementary Table 1), suggesting they can complement each other's activity.

According to the literature, only one protein with 85% or more amino acid identity to one of those described in this article (Dep1627_50) has confirmed capsular activity (Supplementary Figure 5). This is depolymerase from *Klebsiella* phage RCIP0109 (WPJ56835.1) with confirmed KL2 activity (Jing et al., 2026). Overall, the specificity of the phage itself is known in three other cases, while for the others, no information is available in open sources (Supplementary Figure 5). Based on the high conservation of proteins, the capsular activity identified in our experiments can, with a certain degree of probability, be extrapolated to similar proteins with greater than 85% amino acid identity.

While capsular specificity governs initial recognition and adsorption, the therapeutic efficacy of a phage candidate ultimately depends on its replication kinetics, burst size, and capacity to overcome host anti-phage defenses. All four isolated phages exhibited high kinetic efficacy, characterized by short latent periods, alongside exceptional physicochemical stability across broad temperature (4–50 °C) and pH (4–11) ranges. These properties collectively underscore their potential as components of therapeutic cocktails targeting *K. pneumoniae*. Although phages G66 and 1047 demonstrated relatively modest burst sizes (17 and 10 PFU/cell, respectively), which may be suboptimal for monotherapy, such values are consistent with those recently reported for *Klebsiella* phages (Siopi et al., 2024). Moreover, rapid replication cycles in phages may compensate for lower progeny output per infection event (Wang, 2006). Furthermore, burst size is inherently host-dependent; therefore, these phages may yield higher titers when propagated on alternative clinical strains or under different physiological conditions. Consequently, G66 and 1047 remain viable components of multi-phage formulations, where complementary kinetics and receptor specificities can collectively enhance therapeutic efficacy.

The broad environmental stability of these phages offers significant practical advantages, facilitating streamlined storage and transportation while supporting potential efficacy in physiologically variable niches, such as the urinary tract (Żaczek et al., 2020). Further investigation into their performance across diverse *Klebsiella* hosts and in combination with other phages is warranted to fully evaluate their translational potential.

Following genome injection, productive infection can be restricted by bacterial Restriction-Modification (RM) systems, Toxin-Antitoxin (TA) modules, CRISPR-Cas immunity, and other defense mechanisms. RM and TA systems are widespread among *K. pneumoniae* strains and represent a significant barrier to phage infection (Senbadejo et al., 2025). Notably, genomic analysis revealed that all four phages encode putative anti-defense systems (specifically Anti-TA and Anti-RM), which likely enhance their ability to overcome these bacterial barriers. The encoded anti-defense genes may therefore contribute to the observed lytic activity against diverse strains and represent valuable traits for therapeutic applications. Nevertheless, the presence of robust defense systems in certain bacterial strains likely contributes to the observed discrepancies between initial lysis patterns, EOP values, and one-step growth parameters. Indeed, the phages exhibited variable latent periods and burst sizes, reflecting both phage-specific replication strategies and host-dependent intracellular constraints.

## Conclusion

This study reports the isolation and comprehensive characterization of four novel bacteriophage species belonging to the subfamily *Studiervirinae*, recovered from hospital wastewater in Mumbai, India. Phages sourced from this geographically distant reservoir proved effective against antibiotic-resistant *K. pneumoniae* clinical isolates from Russia. Genomic and phenotypic analyses confirmed their strictly lytic lifestyle, robust physicochemical stability, and a favorable safety profile devoid of antimicrobial resistance and virulence determinants. Crucially, these newly described species are equipped with targeted depolymerases, enabling them to effectively breach the capsular barriers of clinically prominent, multidrug-resistant *K. pneumoniae* lineages (KL2, KL22, KL25, KL35, and KL47). Ultimately, these findings support cross-regional environmental screening as a promising strategy for discovering therapeutic phages. Together, these four novel *K. pneumoniae* phage species represent a significant addition to the global therapeutic arsenal against multidrug-resistant pathogens.

## Data Availability

The genome sequences have been deposited in the NCBI GenBank (https://www.ncbi.nlm.nih.gov/genbank/) under the accession numbers PZ481757 (vB_KpnP-SPI_1047), PZ481756 (vB_KpnP-SPI_1627), PZ481758 (vB_KpnP-SPI_KBKp12), and PZ481759 (vB_KpnP-SPI_G66) (BioProject No. PRJNA1469395).
