# Supplementary material for: Characterization of novel Klebsiella pneumoniae phages isolated from hospital wastewater in Mumbai, India
Source: Front Microbiol. 2026 Oct 9;17:1953496. doi: 10.3389/fmicb.2026.1953496 (PMC13691440; doi:10.3389/fmicb.2026.1953496)
Supplement: Supplementary file 1 [file Supplementary_file_1.docx]

**Supplementary Table 1.** Host range, efficiency of plating (EOP), depolymerase activity, and antibiotic resistance profiles of 197 *Klebsiella pneumoniae* strains from the Lopukhin Federal Research and Clinical Center of Physical-Chemical Medicine of Federal Medical Biological Agency and Saint-Petersburg Pasteur Institute’s collections.

**Supplementary Table 2.** Oligonucleotide primers used for cloning of phage-encoded depolymerase genes.

**Supplementary Table 3.** Comprehensive functional annotation of predicted proteins encoded by phages 1047, 1627, KBKp12, and G66.

**Supplementary Figure 1.** SDS‐PAGE showing induced expression and purification of SDS‐PAGE showing induced expression and purification of the depolymerases: **A** – dep1047_48 (88.7 kDa); **B** – dep1627_49 (87.4 kDa) & dep1627_50 (59.1 kDa); **C** – the SUMO-fused protein depKBKp12_46 (92.1 kDa + 14.7 kDa (SUMO) = 106.8 kDa); D – the SUMO-fused protein depG66_49 (62.4 kDa +14.7 kDa (SUMO) = 77.1 kDa). SDS‐PAGE was performed with 10% polyacrylamide gels, 80 V for 30 min, 120‐200V for an hour. Lane 1, proteins profile of *E. coli* BL21 cells before induction; lane 2, proteins profile of *E. coli* BL21 cells after 18 h induction; lane 3, cells lysate; lane 4, supernatant of the cells lysate; lane 5, purified protein; lane 6, marker.

**Supplementary Figure 2.** **A**. Results of spot test for phage 1047 and its depolymerase. 1 – positive control (phage 1047 (10^8^ PFU/ml)); 2 – negative control (elution buffer); 3 – depolymerase dep1047_48; **B**. Results of spot test for phage 1627 and its depolymerases. 1 – positive control (phage 1627 (10^8^ PFU/ml)); 2 – negative control (elution buffer); 3 – depolymerase dep1627_49; 4 – depolymerase dep1627_50. All *Klebsiella pneumoniae* stains are K2-type except EI1939 & EI1940 are K22-type. **C**. Results of spot test for phage KBKp12 and its depolymerase. 1 – positive control (phage KBKp12 (10^8^ PFU/ml)); 2 – negative control (elution buffer); 3 – depolymerase depKBKp12_46.

**Supplementary Figure 3.** The table summarizes depolymerase names, protein lengths and sequences, targeted KL-types. Predicted 3D protein structures generated with AlphaFold3.0 are shown on the right, illustrating domain architecture and the overall structure.

**Supplementary Figure 4.** Attempts to predict structure of the depG66_49 parts. The table shows the amino acid parts of the protein used for structure prediction, the predicted template modeling (pTM) score and the interface predicted template modeling (ipTM).

In addition to determining the KL specificity, structures of proteins were predicted with AlphaFold3.0.

For dep1047_48, the structure is well predicted in the catalytic domain (Supplementary material 3), although the closest depolymerase with a resolved structure (*Klebsiella* phage Kp9 tail fiber gp42; PDB: 7XY1) has less than 50% seq identity.

The proteins responsible for depolymerase activity in phage 1627 are highly conserved within the genus *Przondovirus*. For dep1627_49, the predicted structure is highly likely to be accurate, as *Klebsiella* phage Kp9 tail fiber gp42 (PDB: 7XY1) has 98.46% aa sequence identity. However, the structure is not resolved for the entire protein, but only from 266 aa. The second protein, dep1627_50, is also highly conserved, and its model yields a good per-atom confidence estimate.

Although for depKBKp12_46 there is only one annotated similar protein, the structure is predicted with an acceptable level of confidence. For depG66_49, despite the presence of annotated analogs, the structure clearly cannot be predicted. This is due to the difficulty of obtaining similar proteins from the *Teetrevirus* genus, and there are currently no resolved structures on which AlphaFold could rely. We made attempts to model the protein partial (Supplementary material 4), and modeling starting from amino acid 285 yields a reasonable structure of the enzymatic domain, which is likely close to the natural one.

**Supplementary Figure 5.** Maximum Likelihood phylogenetic trees of identified depolymerases and their closest relatives in amino acid composition from GeneBank. The figure shows the name of the depolymerase source, its GenBank number, length, and amino acid identity with the compared protein. Depolymerases dep1047_48, dep1627_49, dep1627_50 and depG66_49 are marked with a red asterisk. For depolymerase depKBKp12_46 only one similar protein from Klebsiella phage vB_Kpl_K35PH164C3 was found (CAK6597762.1, 99.42% Per. Ident).

It can be noted that for dep1047_48, the same protein from the genetically related *Klebsiella* phage cp8 is missing from the phylogenetic tree (Supplementary material 5). This is because this phage protein is not annotated in GenBank. The *Klebsiella* phage cp8 according to the literature is infected KL10 and KL25 strains [(Lourenço et al., 2023)](https://www.zotero.org/google-docs/?UYIKAs). Describing phage 1047 in our experiments only targets one of these capsule types, KL25. This may be because our sample only included two strains with the KL10 capsule type, and a suitable one was not available. For the remaining dep1047_48-like depolymerases, capsular specificity has not been established.

The same can be said about twenty-nine proteins similar to dep1627_49. Capsule specificity was not described for any of them. KL2 specificity was determined for two phages containing these depolymerases - phages IME183 [(Xu et al., 2023)](https://www.zotero.org/google-docs/?lIDzmq) and vB_KpnP_KpV763 [(Solovieva et al., 2018)](https://www.zotero.org/google-docs/?eqhi8p) - but the enzymes themselves were not tested.

For dep1627_50, even more similar proteins - 44 - have been annotated. Twenty of these belong to the same sources as the proteins similar to dep1627_49. In addition to the already mentioned IME183 and vB_KpnP_KpV763, KL2 activity was demonstrated in phages KP11 [(Huang et al., 2024)](https://www.zotero.org/google-docs/?FJmxbs) and RCIP0109R1 [(Jing et al., 2026)](https://www.zotero.org/google-docs/?iwpVvL). The latter has the ability to infect KL2 and KL111 types, but KL2 activity was demonstrated specifically for the RCIP0109_00047 (WPJ56835.1) protein. Another source of a depolymerase with a similar amino acid composition, the *Klebsiella pneumoniae* phage named phiTH1 has been described in the literature as having activity against the capsule-type KL30 [(Chen et al., 2025)](https://www.zotero.org/google-docs/?KkNpP8), but this activity is demonstrated for a different phage protein (XKB78978.1), not for the dep1627_50-like protein from our list (XKB78977.1).

Capsule specificity has not been established for either the single protein analogous to depKBKp12_46 from the phage vB_Kpl_K35PH164C3, or for the 24 proteins related to depG66_49, or for their parent phages.

Depolymerases from phages infecting other bacterial species can be seen in phylogenetic analysis. This further confirms the presence of horizontal gene transfer. Moreover *Raoultella* is a closely related bacterium that, until 2001, was classified as part of the genus *Klebsiella*.

**References**

[Chen, Y., Fan, Z., Fu, T., Li, Z., Feng, J., Cui, X., Gan, L., Xue, G., Feng, Y., Zhao, H., Cui, J., Yan, C., Xu, Z., Yu, Z., Yang, Y., Ke, Y., & Yuan, J. (2025). Characterization of a Phage-Encoded Depolymerase Against Klebsiella pneumoniae K30 Capsular Type and Its Therapeutic Application in a Murine Model of Aspiration Pneumonia. *Viruses*, *17*(11), 1446. https://doi.org/10.3390/v17111446](https://www.zotero.org/google-docs/?4aRbcF)

[Huang, L., Huang, X., Zhao, T., Zhang, J., & Xiang, Y. (2024). *Isolation and characterization of three lytic podo-bacteriophages with two receptor recognition modules against multidrug-resistant* Klebsiella pneumoniae. Microbiology. https://doi.org/10.1101/2024.05.19.594906](https://www.zotero.org/google-docs/?4aRbcF)

[Jing, S., Song, Y., Bi, X., Song, Y., Wei, D., Huang, J., Zeng, Y., Zhang, G., Zhang, R., Wang, C., & Feng, J. (2026). Programmable *Klebsiella pneumoniae* Phage Tropism Enabled by Scalable Receptor‐Binding Protein Mining and Modular Assembly. *Advanced Science*, *13*(21), e14511. https://doi.org/10.1002/advs.202514511](https://www.zotero.org/google-docs/?4aRbcF)

[Lourenço, M., Osbelt, L., Passet, V., Gravey, F., Megrian, D., Strowig, T., Rodrigues, C., & Brisse, S. (2023). Phages against Noncapsulated Klebsiella pneumoniae: Broader Host range, Slower Resistance. *Microbiology Spectrum*, *11*(4), e04812-22. https://doi.org/10.1128/spectrum.04812-22](https://www.zotero.org/google-docs/?4aRbcF)

[Solovieva, E. V., Myakinina, V. P., Kislichkina, A. A., Krasilnikova, V. M., Verevkin, V. V., Mochalov, V. V., Lev, A. I., Fursova, N. K., & Volozhantsev, N. V. (2018). Comparative genome analysis of novel Podoviruses lytic for hypermucoviscous Klebsiella pneumoniae of K1, K2, and K57 capsular types. *Virus Research*, *243*, 10–18. https://doi.org/10.1016/j.virusres.2017.09.026](https://www.zotero.org/google-docs/?4aRbcF)

[Xu, J., Tian, F., Feng, Q., Li, F., & Tong, Y. (2023). Genomic characterization of the novel bacteriophage IME183, infecting Klebsiella pneumoniae of capsular type K2. *Archives of Virology*, *168*(10), 261. https://doi.org/10.1007/s00705-023-05873-9](https://www.zotero.org/google-docs/?4aRbcF)
